# Revealing in real-time a multistep assembly mechanism for SV40 virus-like particles

**DOI:** 10.1126/sciadv.aaz1639

**Published:** 2020-04-15

**Authors:** Mariska G. M. van Rosmalen, Douwe Kamsma, Andreas S. Biebricher, Chenglei Li, Adam Zlotnick, Wouter H. Roos, Gijs J.L. Wuite

**Affiliations:** 1 Natuur- en Sterrenkunde and LaserLaB, Vrije Universiteit Amsterdam, Boelelaan 1081, 1081 HV Amsterdam, Netherlands.; 2 Department of Molecular and Cellular Biochemistry, Indiana University, 212 S Hawthorne Dr., Bloomington, IN 47405, USA.; 3 Moleculaire Biofysica, Zernike Instituut, Rijksuniversiteit Groningen, Nijenborgh 4, 9747 AG Groningen, Netherlands.

## Abstract

Many viruses use their genome as template for self-assembly into an infectious particle. However, this reaction remains elusive because of the transient nature of intermediate structures. To elucidate this process, optical tweezers and acoustic force spectroscopy are used to follow viral assembly in real time. Using Simian virus 40 (SV40) virus-like particles as model system, we reveal a multistep assembly mechanism. Initially, binding of VP1 pentamers to DNA leads to a significantly decreased persistence length. Moreover, the pentamers seem able to stabilize DNA loops. Next, formation of interpentamer interactions results in intermediate structures with reduced contour length. These structures stabilize into objects that permanently decrease the contour length to a degree consistent with DNA compaction in wild-type SV40. These data indicate that a multistep mechanism leads to fully assembled cross-linked SV40 particles. SV40 is studied as drug delivery system. Our insights can help optimize packaging of therapeutic agents in these particles.

## Teaser

Acoustic force spectroscopy and optical tweezer experiments show a multistep assembly process for SV40 virus–like particles.

## INTRODUCTION

Viruses are remarkable self-assembling systems, which can spontaneously assemble from many individual protein subunits into a closed and ordered capsid structure protecting the viral genome. Simian virus 40 (SV40) is a model virus that can efficiently infect a wide range of human cells. SV40 is shown to be an efficient gene delivery system (*1*–*3*), which makes it an interesting candidate for (targeted) drug delivery approaches. SV40 is a nonenveloped DNA virus belonging to the Polyomaviridae family with a 5.2–kilo–base pair (kbp) circular double-stranded DNA (dsDNA) genome. The native capsid is ~45 nm in diameter (*T* = 7 icosahedral) and consists of three structural proteins, VP1, VP2, and VP3 (*4*, *5*). VP1 is the major capsid protein (CP), which can self-assemble into a virus-like particle (VLP) with *T* = 7 icosahedral symmetry, similar to the native capsid. The major CP stably forms pentamers, from which the five C-terminal arms can lock into neighboring pentamers to form calcium bridges and disulfide linkages, contributing to the capsid stability (*5*, *6*). VP1 is known to bind dsDNA nonspecifically (i.e., independent of sequence) with high affinity via its N-terminal region (*7*). Both RNA and DNA induce in vitro assembly of VP1 pentamers (*8*–*10*). While VP2 and VP3 contribute to the infectivity in vivo (*11*, *12*), they are not necessary for capsid formation. SV40 VLPs with and without VP2/VP3 are extensively studied for usage as drug delivery system (*13*–*17*), and they are morphologically indistinguishable from wild-type (WT) SV40 (*18*).

SV40 VLPs are used as a model system to explore the process of capsid formation. SV40 assembly around dsDNA has been previously studied (*9*, *10*, *19*, *20*), but these studies focused on characterizing the assembly end products. Assembly intermediates could not be sampled, probably because they are too short-lived and instable. Mukherjee *et al.* (*9*, *20*) proposed an assembly model where VP1 pentamers first bind to dsDNA, followed by the formation of protein-protein interactions in which the DNA binding is very fast and the subsequent assembly would be the rate-limiting step. Tsukamoto *et al.* (*10*) suggest that the DNA facilitates capsid assembly by acting as a scaffold determining the shape and size of the assembled structure, which is in agreement with the findings of Kler *et al.* (*8*). However, as none of the experiments were able to follow the assembly process in real time and thus unable to detect possible intermediate states, the actual assembly mechanism of SV40 still remains unconfirmed.

To fill this gap, we use optical tweezers (OT) (*21*, *22*), acoustic force spectroscopy (AFS) (*23*, *24*), and atomic force microscopy (AFM) (*25*–*27*) to explore the mechanism of SV40 assembly around a dsDNA template. We observe fast binding of the VP1 pentamers to the template, which stimulates a first stage of compaction and a significant decrease of the effective DNA persistence length. We suggest that the decrease in persistence length indicates that VP1 pentamers impose kinks in the dsDNA; this is an unexpected means of compacting and packaging nucleic acid in viruses. Next, this kinked DNA seems to be able to form weakly bound loops with the individual pentamers. This is followed by the formation of interpentamer interactions, resulting in semi-stable intermediate SV40 structures that strongly reduce the DNA contour length. Accumulation of these interpentamer and DNA compacting interactions leads eventually to the formation of stable virus-like structures that package a piece of DNA of WT length.

## RESULTS

### DNA binding properties of truncated VP1

To understand the different aspects of the assembly process, we separately studied the DNA protein from the protein-protein (interpentamer) interactions. This allows us to independently study these steps in the assembly process. To this end, we use truncated VP1 pentamers that are missing their C-terminal arms responsible for the major interpentamer interactions but still have the N-terminal motif that binds to dsDNA. In the absence of DNA, the truncated VP1 pentamers do not assemble or aggregate into any virus-like structure, as confirmed by AFM imaging. When incubating truncated VP1 pentamers with DNA (48-kbp λ DNA) under conditions known to induce capsid formation, we observe only individual VP1 pentamers and large unorganized clusters of truncated VP1 pentamers around the DNA (fig. S1). These clusters have a maximal height well below 45 nm, showing that they are unable to form VLPs. This result confirms that the truncated VP1 pentamers are able to bind the DNA but cannot form stable interpentamer bonds. Thus, the truncated VP1 pentamers are ideal to study DNA binding and the biomechanical properties of the VP1 pentamers–DNA complexes, without interference of interpentamer interactions.

**Fig. 1:**
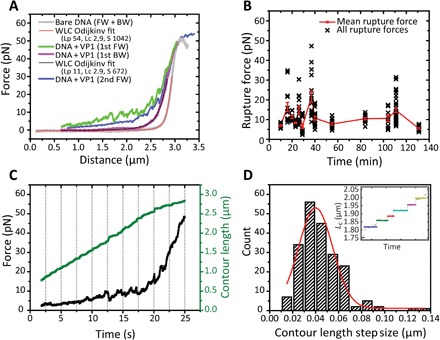
DNA binding properties of truncated VP1 pentamers determined by OT. (**A**) Forward (FW) and backward (BW) FD curves of bare DNA (gray) and DNA incubated with truncated VP1 pentamers for 9 min (green and magenta) and the subsequent immediate restretch (blue). Only backward FD curves are fitted with the WLC model (red and black). (**B**) A scatter plot of all rupture forces (*n* = 190) obtained from 20 DNA molecules in the presence of VP1 (DNA + VP1) after incubation times ranging from 9 to 131 min with the means + SEM for each time point in red. (**C**) An exemplary force-time plot (black) is plotted together with the corresponding change of the *L*_c_ (green). (**D**) Using a step-fitting algorithm, we were able to determine the stepwise lengthening of the *L*_c_ corresponding to the ruptures in our stretching curves (inset). Histogram of all obtained *L*_c_ step sizes; a Gaussian fit to the data (red) shows an average step size of 40 ± 1 nm.

Using OT, we studied the (mechanical) impact of truncated VP1 pentamer binding to pKYB1 dsDNA (8393 bp, 2.8 μm). OT allows us to track the changes in the force-distance (FD) curves as the DNA is stretched. After incubating truncated VP1 pentamers in the presence of DNA for several minutes, we observe a notable increase in the force at short end-to-end distances followed by many small rupture events when we obtain an FD curve (Fig. 1A). When analyzing these rupture events, we do not observe any correlation between the time of incubation and the amplitude of the rupture forces (Fig. 1B). These findings show that truncated VP1 binding to DNA occurs within minutes. The size determination and significance of the rupture events are discussed later.

At forces >40 pN, the FD curve of DNA with truncated VP1 seems similar to the bare DNA curve, suggesting that all DNA-VP1 interactions are broken. However, the backward FD curve of DNA in the presence of VP1 decreases less steeply than the bare DNA backward curve, indicating that the truncated VP1 pentamers are still bound to the DNA. While the experiments in Fig. 1 are performed in buffer solution with free VP1 pentamers present, we repeated these experiments in buffer without free VP1 present. Restretching after relaxation yields a FD curve with the same features as the first stretching curve, confirming that VP1 stays associated with the DNA upon stretching. Through the addition of disassembly buffer, it was possible to remove the VP1 pentamers from the DNA resulting in a stretching curve comparable to the bare DNA stretching curve. This suggests reversible binding of VP1 pentamers to the DNA.

The homogeneous backward FD curves are used to calculate the effective DNA persistence length (*L*_p_) by fitting to the extensible worm-like chain (eWLC) model according to Odijk (*28*) (Fig. 1A). Under our buffer conditions [10 mM Mops (pH 7.2) and 50 mM NaCl], we obtain an effective *L*_p_ for bare DNA of 50 ± 6 nm (*n* = 19), consistent with literature (*29*). For DNA incubated with truncated VP1 pentamers, however, we obtain a drastically lower effective *L*_p_ of 8 ± 1 nm (*n* = 23, all reported errors are SEM unless specified otherwise) independent of incubation time. Furthermore, we also observe a decrease in the stretch modulus *S*, 1000 ± 100 pN for bare DNA and 600 ± 100 pN for DNA in the presence of VP1. The decrease in effective *L*_p_ is most likely due to kinking of the DNA, which allows the assembly reaction to overcome the energetic barrier to tightly bend the DNA and the entropic barrier of forcing it into a small closed container (*30*, *31*).

Next, we quantified the sizes of the observed rupture events. We used the effective *L*_p_ and stretch modulus values deduced from the backward FD curves to calculate the change in contour length (*L*_c_), which corresponds to each measured force point in our FD curve. The resulting *L*_c_ versus time plots (Fig. 1C) were fitted using a step-fitting algorithm based on a change point method (*32*–*34*). This allows us to determine the stepwise lengthening of the *L*_c_ during stretching. We found an average rupture step size of around ~40 nm (Fig. 1D). This is in the order of the *L*_p_ of bare DNA, which hints toward the formation of loops of bare DNA captured by individual VP1 pentamers. This capturing can be explained by the multiple DNA binding sites present on each VP1 pentamer (*5*, *35*). During incubation, these binding sites can compact the DNA by the formation of loops, similar to what has been observed for other DNA compacting agents at low tension (*29*).

**Fig. 2:**
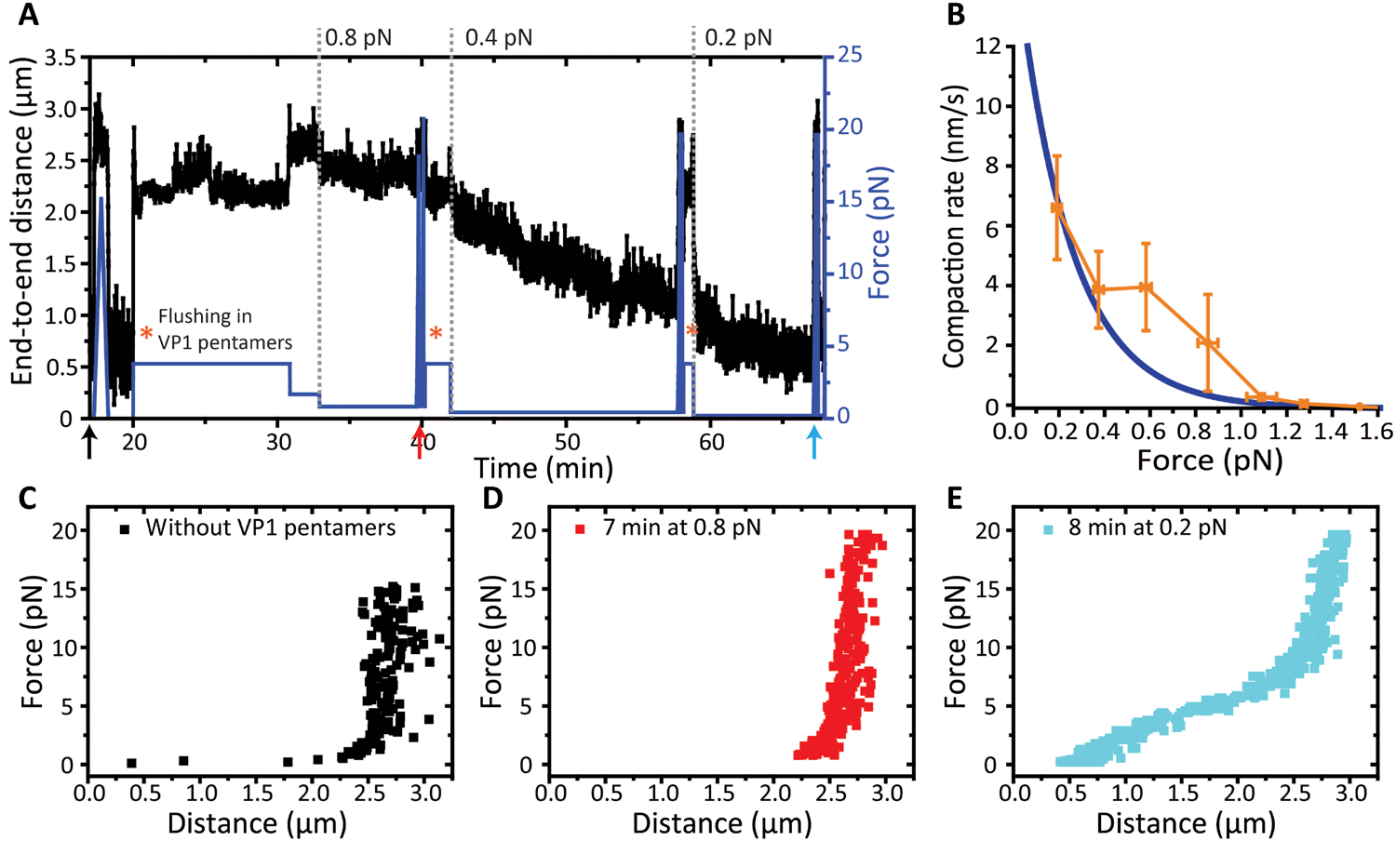
Compaction of DNA by truncated VP1 measured by AFS. (**A**) Applied force and the corresponding end-to-end distance of a DNA molecule plotted over time. Truncated VP1 pentamers are flushed into the flow cell after each force clamp while the DNA is kept at 3.8 pN (orange asterisk). After flushing, the force is lowered to allow VP1 pentamer compaction. (**B**) Average compaction rate measured during clamp cycles at different force values of a total of eight traces. Exponential fit to the data is depicted in dark blue. The compaction rate is underestimated at the lower forces due to the saturation effect at consecutive force clamp cycles (always starting from high force). (**C**) A bare DNA FD curve taken at ~17 min (black arrow) for the molecule shown in (A) (~17 min). (**D** to **E**) FD curves obtained in the presence of VP1 for the molecule shown in (A) taken at ~58 min (red arrow) into ~40 min (blue arrow), respectively.

Next, AFS is used to determine the minimal force that would inhibit compaction of the DNA by the truncated VP1 pentamers. More details on AFS and a schematic representation can be found in the Materials and Methods section. For this experiment, we measured the end-to-end lengths of DNA molecules over time under different tensions in the presence of truncated VP1 (Fig. 2A). Typical FD curves before and during each force clamp cycle are plotted in Fig. 2 (C to E). At low stretching forces, we observe a gradual shortening of the DNA over time, indicating pentamer-induced DNA compaction. We approximated the rate of the induced DNA shortening as a function of the applied force (Fig. 2B). From these data, we can conclude that the truncated VP1 pentamers are only able to bind and compact the DNA when the force is below ~1 pN. The compaction rate increases when lowering the force (Fig. 2B). These observations support our hypothesis that VP1 pentamers are forming loops of DNA as looping will be strongly hindered by force on the DNA. By performing an exponential fit to the data in Fig. 2B, we obtained a characteristic decay force of 0.23 ± 0.02 pN. Using the Boltzmann distribution and *k*_B_*T* ≈ 4 pNnm, yields the distance to the transition state of binding of the VP1 pentamers to the DNA. This is ~17 nm, which is consistent with the formation of a loop of about 40 nm.

### Assembly of VLPs on DNA

Previously, we have shown that incubating WT-VP1 pentamers with λ DNA results in the formation of single VLPs of 44.3 ± 0.3 nm in height (*n* = 112) after 2 hours (*27*). Prolonging the incubation time results in multiple VLPs on a single 48-kbp λ DNA (fig. S1E). This reveals that the DNA can stick out of a fully assembled VLP, of which Tsukamoto *et al*. (*10*) show that the encapsulated part of the DNA is protected from deoxyribonuclease treatment (*10*). The observation that the DNA can stick out a fully assembled VLP is essential because it opens up the possibility to study the assembly of these VLPs in real time while holding the ends of the DNA template in our OT or AFS setups. Using a shorter DNA template, pKYB1, which is only several kilo–base pair longer than WT SV40 DNA, we observe that mostly single VLPs are formed (fig. S1C). Thus, pKYB1 DNA is ideal to study the formation of the assembly of a single VLP with single-molecule methods like OT or AFS.

**Fig. 3:**
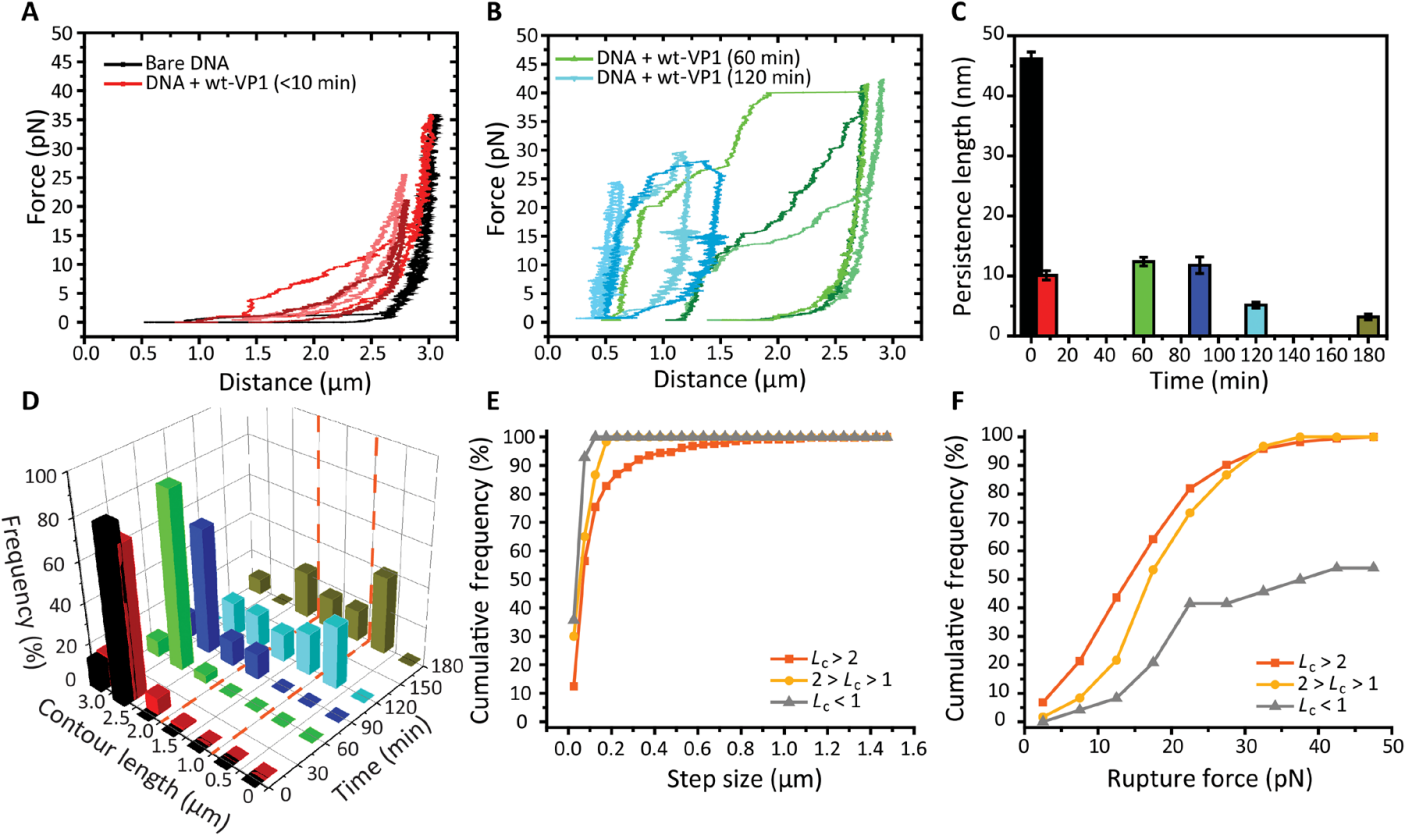
Effect of WT-VP1 pentamer interaction with tethered DNA, followed by AFS in real time. (**A**) Bare DNA FD curves (black) and DNA in the presence of VP1 FD curves obtained <10 min after addition of WT-VP1 pentamers (red; *n* = 3). (**B**) FD curves obtained after incubation for 60 min (green; *n* = 3) or 120 min (blue; *n* = 3). (**C**) Average effective persistence length measured for different incubation times (black, *n* = 123; red, *n* = 97; green, *n* = 25; blue, *n* = 16; light blue, *n* = 29; and brown, *n* = 13; all independent measurements). Error bars represent SEM. (**D**) Three-dimensional plot displaying the frequency of the *L*_c_ as a function of time, same curves as in (C). Bin size is 0.5 μm. (**E**) Cumulative frequency of the rupture step sizes (337, 60, and 14 rupture events were found in 60, 16, and 13 FD curves with a *L*_c_ of >2, between 1 and 2 and <1 μm, respectively). (**F**) Cumulative frequency of the rupture forces from the same data as in (E).

By stretching DNA incubated with WT-VP1 for <10 min in our AFS setup, we observe an early increase in the stretching force and small rupture events (Fig. 3A). This is similar to the results found for the truncated VP1. Note that ruptures appear as horizontal steps because AFS applies a constant force on the DNA; thus, the breakage of a loop leads directly to a lengthening of the tether. We stop our stretching experiments before the overstretching regime (~65 pN) to avoid potential forced dissociation of the pentamers. As before, we fit the backward FD curves with the eWLC and obtain an *L*_p_ of 46 ± 1 nm for bare DNA. The effective *L*_p_ decreases to 10.0 ± 0.1 nm within 10 min after the addition of WT-VP1 (Fig. 3C). The WT-VP1 thus seems to bind and kink the DNA in a similar fashion and speed as the truncated VP1.

Notably, we observe that the step sizes corresponding to the rupture events in case of the WT-VP1 pentamers increases in size to an average of 100 ± 10 nm within 10 min compared to the 40 ± 1–nm ruptures obtained with the truncated VP1 pentamers (fig. S2A). Presumably, these larger steps are due to pentamers that are starting to interact with one another in addition to loops being formed within a single pentamer. The forces, however, at which the ruptures occur for the WT-VP1 pentamers incubated for <10 min, are comparable as previously observed for the truncated VP1 pentamers, 12.0 ± 0.4 pN and 11 ± 1 pN, respectively. So, the initial interaction between the pentamers is rather weak. However, the rupture force obtained for the WT-VP1 pentamers increases to 15 ± 1 pN and 19 ± 1 pN after 60 and 90 min, respectively (fig. 2B). This increase is in line with the possibility that disulfide cross-links can be formed between pentamers (*6*). Thus, we believe that the higher rupture forces and larger step sizes are observed because of the formation of interpentamer, potentially cross-linked, interactions, i.e., the formation of intermediate structures.

Another clear observation in these experiments is a strong reduction in the *L*_c_ over time. To quantify this, we determine the *L*_c_ from the backward FD curves (Fig. 3E). We also find differences in *L*_c_ obtained at the same incubation time, which would indicate that different structures form within a similar time frame. Therefore, we decide to analyze the data with respect to their *L*_c_, i.e., compare similar structures with each other independent of incubation time (Fig. 3, E and F). DNA molecules with an *L*_c_ of >2 μm show relative low rupture forces with large step sizes and a shoulder-like stretching behavior (fig. 3A) suggestive for weak or no interpentamer interactions (*36*). In curves with decreased *L*_c_ (*L*_c_ <2 μm), we observe a more linear stretching behavior, which indicates the formation of intermediate structures due to strong interpentamer interactions (*36*).

The molecules that show a decrease of the *L*_c_ of <2 μm seem to naturally fall in two categories: molecules with a *L*_c_ between 1 and 2 μm and molecules with a *L*_c_ of <1 μm. The molecules with a *L*_c_ between 1 and 2 μm display rupture forces that are larger and rupture step sizes that are significantly smaller compared to the group with a *L*_c_ of >2 μm. This indicates that more and stronger interpentamer interactions are formed as the result of intermediate structure formation. The group of molecules with a *L*_c_ of <1 μm only displays few and small rupture step sizes. The rupture forces are higher than observed for the molecules with a *L*_c_ of >1 μm. In addition, the total number of rupture forces decreases with decreasing *L*_c_ of 3.8 ruptures per DNA with *L*_c_ between 1 and 2 μm and 1.1 ruptures per DNA molecule with *L*_c_ of <1 μm (fig. S4). Thus, it seems that with decreasing *L*_c_, the number of ruptures decreases, the step size decreases, and the average rupture force increases, which suggests that more stable structures are formed.

About half of the molecules with an *L*_c_ of <1 μm do not show any rupture event up to an average stretching force of 24 ± 1 pN (fig S3). This would be an indication for the formation of a complete VLP that, once it reached its fully formed icosahedral structure, will have an increased stability (*37*, *38*). A fully formed VLP would also be a ready explanation for the drastic length reduction that is observed in these cases: A measured *L*_c_ of ~1 μm corresponds to a shortening of 5500 bp, which is close to the size of the WT SV40 genome (5.2 kbp). In addition, only a single VLP would fit on our pKYB1 DNA template (8393 bp), and the time frame of incubation (120 to 180 min) matches with the formation of a single VLP on DNA as shown with our AFM experiments (*27*). Hence, these four lines of evidence are a strong indication that the most stable structures we find are fully formed, cross-linked VLPs assembled on our dsDNA template.

## DISCUSSION

**Fig. 4:**
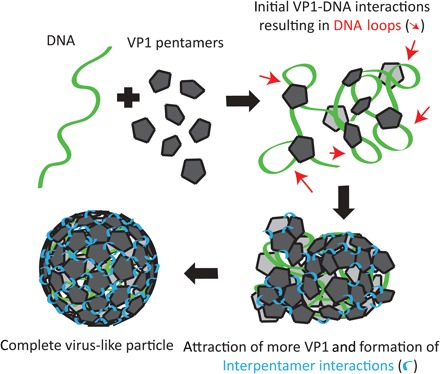
Schematic representation of our assembly model. When DNA (green) is incubated with VP1 pentamers (gray), the VP1 pentamers will immediately bind to the DNA followed by compaction of the DNA as a result of looping (indicated with red arrows). Binding of VP1 to DNA raises the local concentration of VP1 pentamers, which allows the formation of interpentamer interactions (indicated in blue). Over time, these interactions become stronger possibly by the formation of disulfide bridges. Last, this results in the formation of a complete stable VLP.

Different mechanisms of virus assembly around a nucleic acid substrate can be distinguished according to literature (*35*, *37*, *39*). Our OT, AFS, and AFM observations are in agreement with the proposed disordered pathway mechanism where the CP bind the genomic template and are then rearranged into a capsid. This pathway is associated with weak CP-CP interactions in relation to CP-genome interactions. We observed this two-step pathway, yet we reveal additional mechanisms that lead to the formation of a fully formed capsid. The assembly mechanism we propose for SV40 is the following: VP1 pentamers rapidly bind to the DNA, which raises the local concentration of VP1 pentamers, i.e., DNA act as an antenna (*40*). We show that binding also results in a much smaller effective *L*_p_ most likely due to kinking of the DNA. In addition, we observe that the DNA gets further compacted as the result of loop formation between the multiple DNA binding sites of each VP1 pentamer (*5*). In addition, this compaction will further increase the local pentamer concentration, resulting in more potential interpentamer interactions. Next, interpentamer interactions lead to the formation of increasingly large intermediate structures where over time the weak interpentamers interactions likely stabilize due to the formation of disulfide bridges. The VP1 pentamers will keep rearranging themselves to form the energetically most favorable state, a VLP where interpentamer interactions are too strong to break with our stretching forces (fig. S3). A schematic representation of the assembly mechanism is shown in Fig. 4.

A disordered assembly pathway is previously found for other viruses, such as, for instance, for the RNA virus cowpea chlorotic mottle virus (CCMV) (*41*). Assembling SV40 CP on an RNA template results in a different pathway, a high-cooperativity pathway leading to the formation of ~22-nm VLPs (*42*). In general, it seems that there is a trade-off between the stiffness of the polymer to be encapsulated and the strength of the protein-protein interactions (*35*, *37*, *39*). CCMV has, for instance, a preference for packaging of polystyrene sulfonate (PSS), which favors a collapsed state over single-stranded RNA, which acts like a many-branched polymer (*43*). For SV40, it is shown that tertiary structure of PSS or RNA and the size of the substrate affect the assembly (*19*). All these experiments show that the template around which the capsid forms decisively influences assembly.

In our experiments, we stay close to the WT genome of SV40 as we use a dsDNA template for assembly leading to 45-nm VLPs similar to WT capsids. The multistep SV40 assembly mechanism we propose is in agreement with the observations of Mukherjee *et al* (*9*, *20*) and Tsukamoto *et al* (*10*); however, the disordered pathway we reveal displays additional, unreported mechanisms that are not necessarily sequential. The described experimental approach based on OT and AFS could be generally applicable to study viral assembly around a template in real time. The multistep assembly mechanism we revealed here provides insight in different molecular dynamics that lead to the formation of an infectious particles and thus might guide the optimization of the production of SV40-based VLPs for therapeutic applications.

## MATERIALS AND METHODS

### SV40 VP1 VLPs

SV40 VP1 VLPs were obtained from *Spodoptera frugiperda* (Sf9) cells as described in (*20*, *27*). SV40 VP1 VLPs were dissociated into VP1 pentamers by the addition of 20 mM tris-Cl (pH 8.9), 5 mM EDTA, and 2 mM dithiothreitol (disassembly buffer). After 30 min, the sample was imaged with AFM to confirm complete dissociation (fig. S1, A and B). Buffer was exchanged to assembly buffer, 10 to 50 mM Mops (pH 7.2) and 50 to 125 mM NaCl using Amicon Ultracentrifugal filters 100k (Millipore, Burlington, MA). The absence of the C-terminal arms was checked with agarose gel electrophoresis, and a 5-kDa band shift was observed in case of truncated VP1.

### Atomic force microscopy

Truncated VP1 pentamers were incubated with λ DNA (48,502 bp; Roche Diagnostics GmbH, Mannheim, Germany) for 3 hours at room temperature at a ratio of 2750:1 before immobilization onto a hydrophobic glass slide as previously described (*44*). This high ratio is due to difficulties with adhering the truncated VP1 pentamer-DNA structures to the hydrophobic glass surface [and aminopropyltriethoxysilane (APTES)–coated mica]. WT-VP1 pentamers were incubated overnight at room temperature with λ DNA or pKYB1 at a ratio of 360:1 and immobilized onto an APTES-coated mica surface. Freshly cleaved mica was incubated for 80 s in a 1% APTES in ethanol solution and rinsed in succession with acetone, 70% ethanol, and milliQ water. Both samples were incubated at a volume of 40 to 100 μl for approximately 15 min at room temperature before imaging. Imaging of the viral structures was performed with an AFM from Nanotec Electronica (Madrid, Spain), operated in jumping mode (*45*). Olympus (Tokyo, Japan) OMCL-RC800PSA rectangular, silicon-nitride cantilevers with a nominal tip radius of 15 nm, and a nominal spring constant of 0.05 N/m were used. The obtained images were processed using the WSxM software.

### DNA tethering

DNA tethers for OT and AFS were made, as described elsewhere with some minor changes (*23*). The flow cell (LUMICKS B.V., Amsterdam, The Netherlands) was cleaned with bleach and treated with 500 nM sodium thiosulfate and thoroughly rinsed with phosphate-buffered saline (PBS) before each measurement. The flow cell was incubated with anti-Dig antibody-containing solution (20 μg/ml; Roche) in PBS for 20 min. A two-step passivation was used, 30 min in bovine serum albumin [0.2% (w/v); Sigma-Aldrich, Saint Louis, MO) and 30 min in pluronic [0.5% (w/v), F127], both in PBS. Thereafter, buffer containing the biotinylated/digoxigenylated pKYB1 DNA (expressed in *Escherichia coli*, vector of 8393 bp) was incubated for 20 min. Last, streptavidin-coated polystyrene microspheres (Spherotech, Lake Forest, IL) were incubated for 20 min.

### Optical tweezers

The OT measurements were conducted with a previously described instrument (*46*). We used either a single or double OT to capture, manipulate, and measure the force on the DNA/DNA-protein construct. In the single-OT experiments, one end is fixed to a streptavidin-coated surface and the other end is held by a streptavidin-coated bead (1.84 μm; Spherotech) kept in the trap. Between the two tethering points, a single DNA molecule with biotinylated ends was tethered. For high-resolution force, we used back focal plane interferometry, meaning that the interference signal of the laser beam with light scattered from the microsphere is measured. We incubated the DNA under the following conditions: 100 nM truncated VP1 pentamers in 10 mM Mops (pH 7.2) and 50 mM NaCl at room temperature. The average pulling rate was approximately 2 pN/s. Data analysis was performed using custom-written software in LabView (National Instruments) and OriginPro (OriginLab).

### Acoustic force spectroscopy

**Fig. 5:**
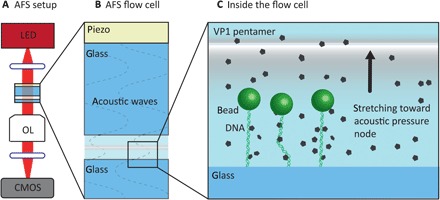
Schematic representation of the AFS setup and the inside of the flow cell (not to scale). (**A**) The flow cell is imaged using an inverted microscope with objective lens (OL), a digital camera [complementary metal-oxide semiconductor (CMOS)] and light-emitting diode (LED) light source (455 nm). (**B**) The flow cell itself consists of two glass plates, which have a fluid chamber in between. A transparent acoustic wave generating piezo element is attached to the upper glass plate, which is electronically connected to control and measure the voltage. (**C**) In the flow cell, DNA molecules of which one side is attached to the glass and the other to a microsphere (bead) are stretched toward the acoustic pressure node by acoustic forces acting on the microsphere. Figure inspired by (*23*, *24*).

For AFS experiments, we used pKYB1 DNA in combination with 4.5-μm beads to make DNA tethers. A schematic representation of the AFS setup can be found in Fig. 5. The following conditions were used: 100 nM truncated VP1 pentamers in 10 mM Mops (pH 7.2) and 50 mM NaCl at room temperature. For assembly experiments, we used 330 to 360 nM freshly prepared WT-VP1 pentamers in 50 mM Mops (pH 7.2), 125 mM NaCl, and 2 mM CaCl_2_ at room temperature. In parallel, we always performed the in vitro assembly reaction on DNA after which we imaged by AFM at a similar time frame to confirm protein activity and its ability to form VLPs. A detailed description of the AFS instrumentation can be found elsewhere (*23*, *24*).

### Analysis

We used the eWLC model (*28*) to approximate the DNA properties according to

$$x=L_{c}[1-\frac{1}{2}{\left(\frac{k_{B}T}{{FL}_{p}}\right)}^{\frac{1}{2}}+\frac{F}{S}]$$

where *x* is the extension (end-to-end distance) of the DNA, *L*_c_ is the contour length, *k*_B_*T* is Boltzmann’s constant times absolute temperature, *F* is the force, *L*_p_ the persistence length, and *S* the stretch modulus of DNA. An elsewhere-described (*29*) home-built MATLAB program was used for fitting of our stretching curves. In case of truncated VP1 pentamers with DNA, the *L*_c_ was fixed since these VP1 pentamers are not able to compact the DNA.

To determine the DNA shortening over time and the rupture forces and corresponding step size of the rupture events observed in our stretching curves, we used a home-built MATLAB program based on the change point method. Segments will be fitted along the stretching curves, and these segments can either have a fixed or a variable slope. The change point method uses the likelihood ratio to evaluate whether to start a new segment, and this will be determined by a given critical value. A more detailed description of the change point method can be found in (*32*–*34*).

## Supplementary Material

aaz1639_SM.pdf

## SUPPLEMENTARY MATERIALS

Supplementary Materials for this article is available at http://advances.sciencemag.org/cgi/content/full/6/16/eaaz1639/DC1

## Appendix

View/request a protocol for this paper from *Bio-protocol*.
