# Supplementary material for: Revealing in real-time a multistep assembly mechanism for SV40 virus-like particles
Source: Sci Adv. 2020 Apr 15;6(16):eaaz1639. doi: 10.1126/sciadv.aaz1639 (PMC7159915; doi:10.1126/sciadv.aaz1639)
Supplement: aaz1639_SM.pdf [file aaz1639_SM.pdf]

Science Advances
AAAS

advances.sciencemag.org/cgi/content/full/6/16/eaaz1639/DC1

Supplementary Materials for

Revealing in real-time a multistep assembly mechanism for SV40 virus-like particles

Mariska G. M. van Rosmalen, Douwe Kamsma, Andreas S. Biebricher, Chenglei Li, Adam Zlotnick, Wouter H. Roos*, Gijs J.L. Wuite*

*Corresponding author. Email: g.j.l.wuite@vu.nl (G.J.L.W.); w.h.roos@rug.nl (W.H.R.)

Published 15 April 2020, *Sci. Adv.* **6**, eaaz1639 (2020)
DOI: 10.1126/sciadv.aaz1639

This PDF file includes:

Figs. S1 to S4

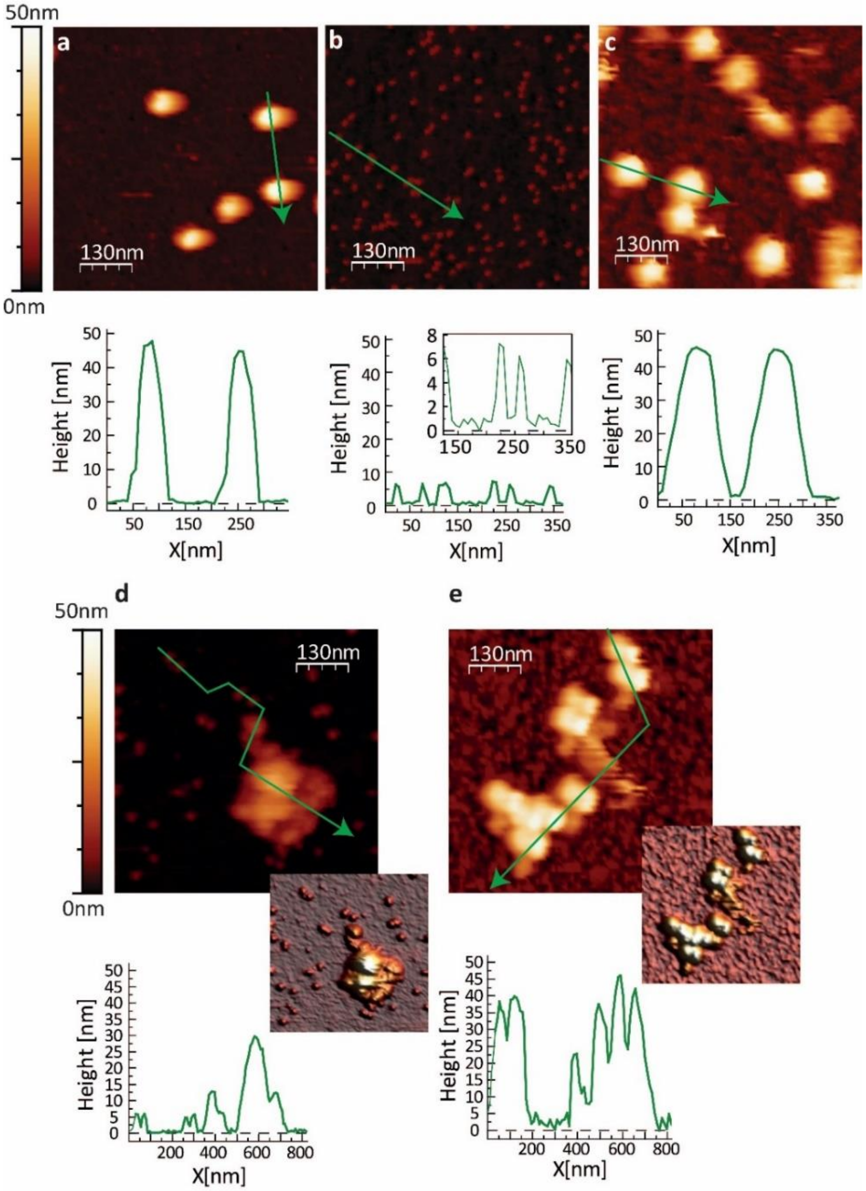

Fig. S1. AFM data of different VP1 pentamer assemblies. a-c) Topographical 2D AFM images with height profile alone the green line. a) SV40 VLPs that are immobilized on hydrophobic glass. The VLPs are round with a height of 45 nm. b) VP1 pentamers with a height of approximately 6 nm were obtained from VLPs by the addition of the disassembly buffer components 5 mM DTT and 5 mM EDTA in a low ionic strength buffer with pH 8.9. c) Overnight incubation of wt-VP1 pentamers with pKYB1 DNA results in the formation of multiple VLPs of ~45 nm. d-e) Topographical 2D and 3D image together with a height profile along the green arrow. d) When incubating truncated VP1 pentamers with lambda DNA for ~3 hours, large unorganized clusters of DNA-VP1 pentamers with a maximum height of 25-30 nm and an irregular shape were observed. e) Overnight incubation of wt-VP1 pentamers with lambda DNA results in the formation of multiple VLPs on 1 or more DNA molecules in a ‘beads-on-a-string’ like formation. A height of about 45 nm is in agreement with previous papers also studying the assembly of SV40 VLPs [9, 10, 18].

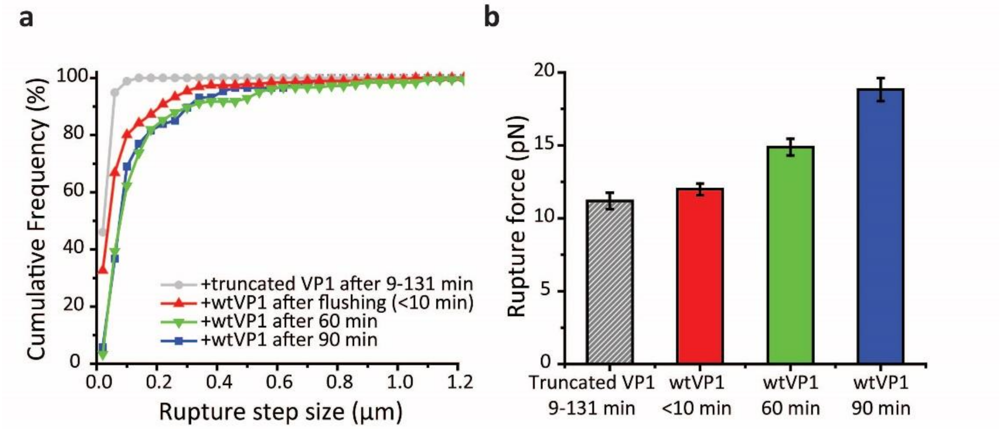

Fig. S2. Quantification of the rupture events during FD curves. a) Cumulative frequency of the rupture step sizes obtained with truncated VP1 pentamers and wt-VP1 pentamers. b) Average rupture force obtained from the forward stretching curves of DNA incubated with truncated VP1 pentamers and wt-VP1 pentamers. A total number of 176, 231, 183 and 87 rupture events were found for 20, 97, 25 and 16 different DNA molecules with truncated VP1 pentamers (incubated up to 131 min) or wt-VP1 pentamers (incubated for <10, 60 and 90 min), respectively. The average pulling rate was approximately 2 pN/sec. Error bars represent standard error of the mean.

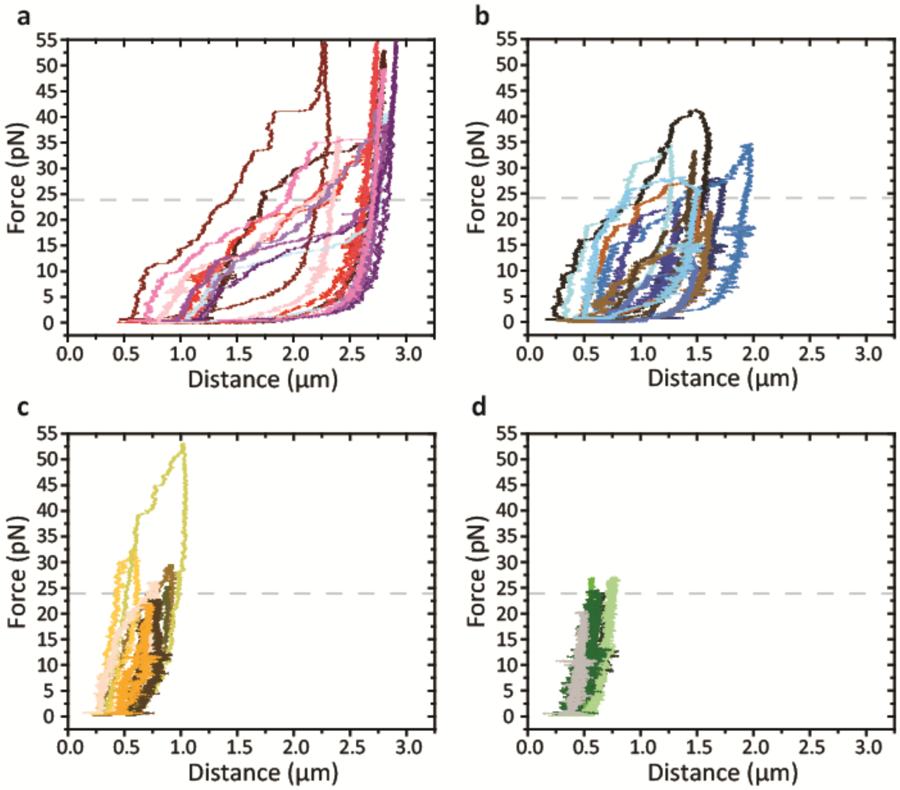

Fig. S3. Exemplary FD curves obtained at time points between 60 – 180 min. a-d) Ten representative FD curves with an obtained Lc of >2 (a), between 1 and 2 (b) and <1 with (c) and without (d) rupture events. The dotted line is drawn at a force of 24 pN (which is the average maximal stretch force of the group with Lc <1 µm without rupture events) to more clearly show the difference in hysteresis, due to rupture events, between the groups.

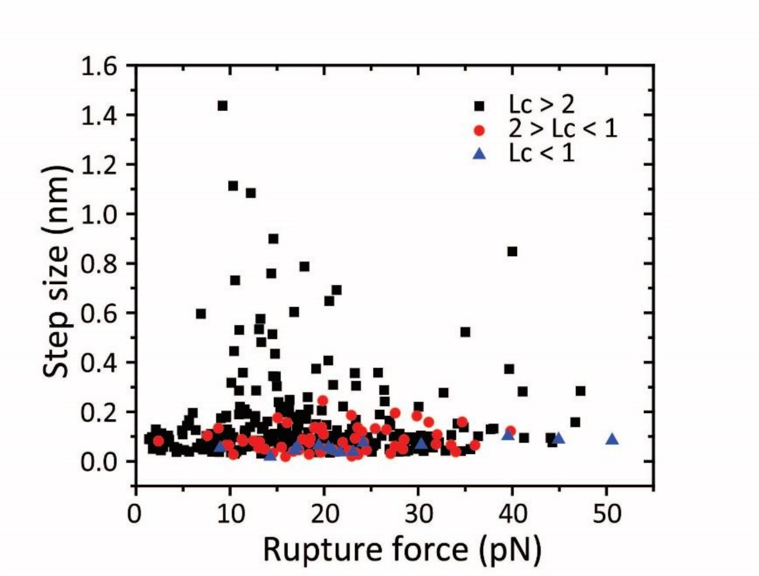

Fig. S4. Scatter plot of the rupture force versus step size of all ruptures found (337, 60 and 14 rupture events were found in 60, 16 and 13 FD curves with a Lc of >2, between 1 and 2 and <1 µm, respectively resulting in an average number of ruptures/DNA molecule of 5.6, 3.8 and 1.1 respectively).
